# Contralateral improvement of cerebrovascular reactivity and TIA frequency after unilateral revascularization surgery in moyamoya vasculopathy

**DOI:** 10.1016/j.nicl.2021.102684

**Published:** 2021-04-21

**Authors:** Pieter T. Deckers, Wytse van Hoek, Annick Kronenburg, Maqsood Yaqub, Jeroen C.W. Siero, Alex A. Bhogal, Bart N.M. van Berckel, Albert van der Zwan, Kees P.J. Braun

**Affiliations:** a Department of Neurology and Neurosurgery, UMC Utrecht Brain Center, Utrecht, the Netherlands; b Department of Radiology and Nuclear Medicine, Amsterdam UMC, Location VUmc, Amsterdam, the Netherlands; c Imaging Division, Department of Radiology, Utrecht Center for Image Sciences, University Medical Center Utrecht, Utrecht the Netherlands; d Spinoza Centre for Neuroimaging, Amsterdam, the Netherlands

**Keywords:** PET, Cerebrovascular reactivity, Moyamoya, Revascularization, TIA

## Abstract

**Objective:**

Moyamoya vasculopathy is a rare, often bilateral disease characterized by progressive stenosis and occlusion of the distal internal carotid artery, leading to a progressive deterioration of cerebrovascular reactivity (CVR) and increased risk of transient ischemic attacks (TIAs), infarction and hemorrhage. Surgical revascularization is a widely accepted symptomatic treatment, often performed bilaterally in one or two stages. To possibly further optimize treatment strategy, we investigated the effect of unilateral revascularization surgery on the CVR of, and TIA frequency originating from, the contralateral hemisphere.

**Methods:**

From our database of 143 moyamoya vasculopathy patients we selected those with bilateral disease, who underwent hemodynamic imaging ([15O]H2O positron emission tomography (PET)-CT with acetazolamide challenge) before and 14 months (median) after unilateral revascularization. We evaluated CVR in three regions per hemisphere, and averaged these per hemisphere for statistical comparison. Conservatively treated patients were showed as a comparison group. To examine TIA frequency, we selected patients who presented with TIAs that (also) originated from the contralateral – not to be operated – hemisphere. We scored changes in CVR and TIA frequency of the ipsilateral and contralateral hemisphere over time.

**Results:**

Seven surgical and seven conservative patients were included for CVR comparison. Of the 20 scored contralateral regions in the surgical group, 15 showed improved CVR after unilateral revascularization, while 5 remained stable. The averaged scores improved significantly for both hemispheres. In conservatively treated patients, however, only 3 of the 20 scored regions improved in the least-affected (contralateral) hemispheres, and 9 deteriorated. From the 6 patients with contralateral TIAs at presentation, 4 had a decreased TIA frequency originating from the contralateral hemisphere after unilateral surgery, while 2 patients remained stable.

**Conclusion:**

Both CVR and TIA frequency in the contralateral hemisphere can improve after unilateral revascularization surgery in bilateral MMV.

## Highlights

• Contralateral cerebrovascular reactivity may improve after unilateral surgery in moyamoya.
• TIA frequency from the contralateral hemisphere can decrease after unilateral moyamoya surgery.
• These findings support staged rather than direct bilateral surgery in moyamoya.

## 1 Introduction

Moyamoya Vasculopathy (MMV) is a rare disease characterized by usually bilateral progressive stenosis or occlusion of the distal internal carotid artery and its branches. This may cause regional hypoperfusion, which can lead to transient ischemic attacks (TIAs), cerebral infarction and cognitive decline (Scott and Smith, 2009). Newly formed reactive collaterals – so called “moyamoya vessels” - serve to improve brain perfusion but may also predispose to brain hemorrhage. When left untreated, MMV may cause overall clinical deterioration as the result of chronic hypoperfusion, infarction or hemorrhages (Kronenburg et al., 2018).

Although there is no cure for the disease, revascularization surgery – consisting of either direct bypass surgery or indirect procedures to promote neovascularization – is a widely accepted symptomatic and preventative treatment (Jeon et al., 2018, Pandey and Steinberg, 2011). Revascularization poses certain risks, e.g. *peri*-operative infarction, hemorrhage and hyperperfusion syndrome (Kronenburg et al., 2014, Yu et al., 2016). For surgical decision making and planning, most centers perform extensive preoperative evaluations. This includes: digital subtracting angiography ((DSA) to assess the severity of the arteriopathy and the presence and extent of collateral networks) and some form of cerebrovascular hemodynamic measurements. The latter is used to determine the indication for, and localization of, surgery and to evaluate the rate of progression of the disorder. Cerebral blood flow (CBF) and cerebrovascular reactivity (CVR: the ability of vessels to dilate and subsequently regulate CBF) are the most essential hemodynamic parameters (Fierstra et al., 2018, Pandey and Steinberg, 2011). For CVR assessment [^15^O]H_2_O positron emission tomography (PET) in combination with a vasodilatory challenge (e.g. acetazolamide or CO_2_ inhalation) is often considered the gold standard (Fierstra et al., 2018, Raichle et al., 1983). Contrary to healthy subjects, the CBF of MMV patients does not always increase after a vasodilatory challenge with ACZ. In some affected regions there is already maximal vasodilation to compensate for the CBF reduction. In these regions, CVR after a challenge is absent or even negative. This paradoxical decrease of CBF is the so-called steal phenomenon which is strongly associated with risk of infarction (McKetton et al., 2019, Webster et al., 1995, Yonas et al., 1993) and cortical thinning (Fierstra et al., 2011, Fierstra et al., 2010). The presence of steal can be one of the reasons to decide to surgically treat a patient (Mandell et al., 2011). After revascularization, extra-cranial to intracranial blood flow improves perfusion and partially restores CVR; thus, reducing the risks of TIAs and infarctions (Jeon et al., 2018).

In bilateral MMV the revascularization is usually performed on both sides. This can either be done in two separate sessions (as is customary in our institution), starting with the most symptomatic or hemodynamically affected hemisphere or by operating both hemispheres in a one-stage approach (Czabanka et al., 2016, Khan et al., 2003). In the preoperative DSA, shunting of blood may be seen from the least affected hemisphere to the most affected hemisphere, e.g. through the anterior communicating artery or via leptomeningeal collaterals (Lim et al., 2007). Theoretically, this could mean that by improving the blood flow unilaterally, less shunting is required and perfusion of both hemispheres improves, with a reduced risk of steal in the contralateral hemisphere. This would imply that a second operation is not always necessary, even though initially considered mandatory. In these patients, avoiding a second operation could considerably reduce the risks and treatment burden.

In our experience, it is common that patients with bilateral MMV who have undergone unilateral surgery show an improvement of contralateral CBF and CVR and a decrease in TIA frequency originating from the unoperated hemisphere. With this study, we aim to systematically investigate the effect of a unilateral first surgical procedure on the CVR and TIA frequency of the contralateral hemisphere.

## 2 Methods

### 2.1 Patient selection

For this retrospective observational cohort study of patients with bilateral MMV treated in our center, the Dutch Medical Research Involving Human Subject did not apply and informed consent was not required. We used an existing MMV database, consisting of patients with MMD (moyamoya disease, as defined by the criteria posed by the Research Committee on the Pathology and Treatment of Spontaneous Occlusion of the Circle of Willis (2012) and MMS (moyamoya syndrome (Scott and Smith, 2009)). For the study of postoperative contralateral CVR changes we selected bilaterally affected patients from this database of whom PET scans (with ACZ challenge) before and after a unilateral operation were available, or those who were treated conservatively and had at least two PET scans available with an interval of at least one year. We excluded patients who received any neurosurgical treatment before being presented at our center. Data of patients who received a conservative treatment (wait and carefully follow policy) first, and were operatively treated in a later stage of their disease, and who had two PET scans during the conservative treatment stage and also PET scans before and after surgical treatment, were included twice; both in the surgery group and in the conservative treatment group.

**Fig. 1:**
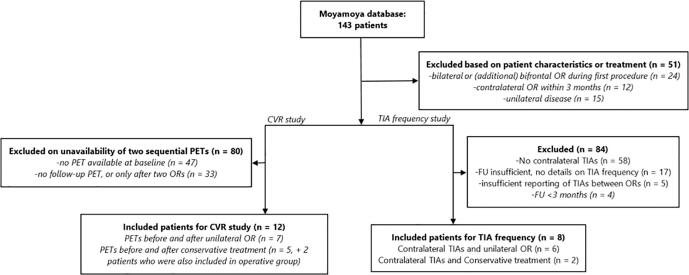
Flowchart of patient inclusion. (abbr.: OR: operation, CVR: cerebrovascular reactivity, FU: follow-up).

For the study of postoperative changes in contralateral TIA frequency, we selected patients who were unilaterally treated and also had TIAs originating from the contralateral (unoperated) hemisphere at presentation. We excluded patients if clinical follow-up data were not available, or follow-up duration was less than three months after the first operation (thus preventing reasonable assessment of clinical improvement). Patients who received a contralateral operation within three months after the first procedure were also excluded. See the flowchart in Fig. 1 for the full details of patient selection. For all included patients, we noted whether or not new TIAs, infarctions or hemorrhages had occurred at follow-up.

### 2.2 Treatment

All patients were treated with acetylsalicylic acid (100 mg for adults, 38 mg for children) and all children were advised to increase their fluid intake. In our center, ultimately the decision to treat surgically is left to the discretion of the treating physician, but in general revascularization is indicated if patients are clinically symptomatic, or if the PET reveals severely compromised CVR. Children with bilateral MMV first undergo revascularization of the most affected hemisphere, consisting of a combination of a direct STA-MCA bypass (if possible) and a local encephalo-duro-myo-synangiosis (EDMS). In some patients, additional bifrontal encephalo-duro-periosteal-syngiosis (EDPS) was performed (described elsewhere; (Esposito et al., 2015) patients receiving additional bifrontal EDPS could not be included in the current study). For adult patients the preferred operative treatment was a unilateral STA-MCA bypass with EDMS only. If an operation was not directly considered indicated – e.g. in case of stable disease course with no recent ischemic events or relatively spared CVR - patients were closely monitored and followed over time.

### 2.3 PET acquisition and analysis

Semi-quantitative [^15^O]H_2_O PET (Siemens HR + and Gemini TF‐64 PET/CT (Philips Medical Systems, Cleveland, OH, USA)) was performed (as described elsewhere (Heijtel et al., 2014), without arterial sampling in almost all patients who were diagnosed with MMV. Briefly summarized: the protocol started with a low dose CT (PET-CT) or a 10-min transmission scan (HR + scanner) for attenuation purposes. A bolus of approximately 550 MBq [^15^O]H_2_O was injected, simultaneously starting a 3D dynamic emission scan. Raw data were reconstruction into 25 frames (frame durations: 15, 6x5, 3x10, 3x15, 3x20, 4x30, 5x60s). Only summed images from 15 to 105 s were used and corrected for injected dose and the decay was corrected for differences in exact start scan time and dose calibration time, generating standardized uptake images for visual inspection. Each study consisted of a two 10 min PET emission scans, one before and one after intravenous administration of 20 mg/kg acetazolamide (max dose 1 g.). After careful coregistration on basis of the low dose CT images, a subtraction image was created by subtracting the pre ACZ image from the post ACZ image.

If surgery was performed, most patients underwent postoperatively the same PET procedure, after a mean interval of 14 months. For those treated conservatively, the PET scans were often – but not always – repeated, to monitor disease progression.

**Fig. 2:**
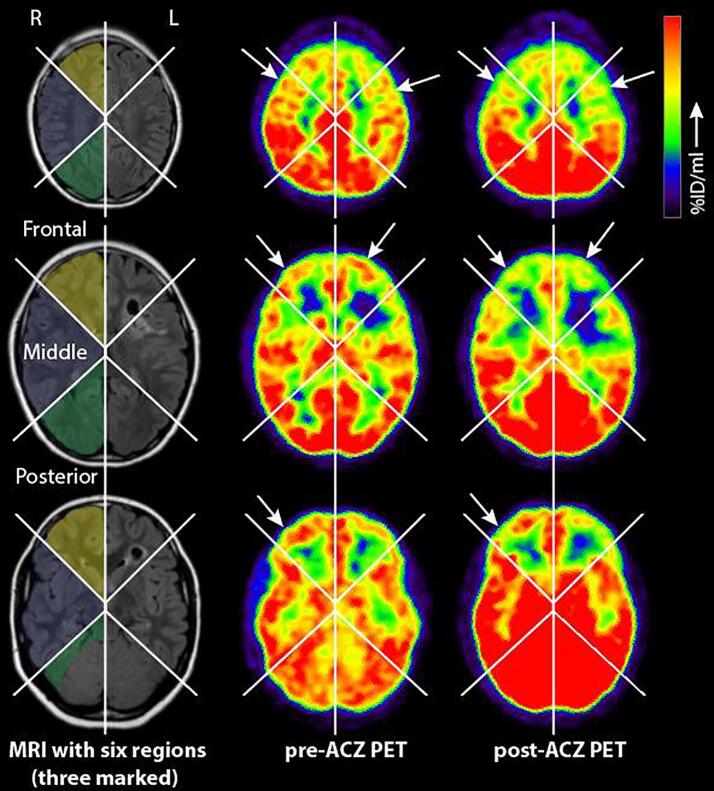
Example of six investigated regions and PET CVR scores. The baseline T2-FLAIR weighted MRI, pre-ACZ and post-ACZ PET images of the same patient shown at three slices (for the PET CVR score all available slices were used). The first column shows the six different regions of interest drawn onto the T2-FLAIR, with the three right regions colored for clarity (yellow: frontal regions, blue: middle regions, red: posterior region. Note: the cerebellum is not part of the posterior region, but is used as a reference). The middle and last columns show the pre- and post-ACZ PET results. The arrows show some examples of (parts of) regions that show vascular steal (negative CVR). The CVR-score of the preoperative scan of this patient is: “steal” in bilateral frontal, and middle territories and “normal reactivity” in bilateral posterior regions. The patient had an infarction left frontally as seen in the T2-flair images. Abbreviations: ACZ: acetazolamide, PET: Positron Emission Tomography, %ID/ml: percentage injected dose per milliliter. (For interpretation of the references to color in this figure legend, the reader is referred to the web version of this article.)

The perfusion images from the PET were visually scored by two blinded reviewers (PTD, AK) at the same time. When in doubt, a consensus meeting was planned with the senior author (KB). For scoring, all slices of the perfusion images were divided into six regions: a frontal, middle and posterior region for both hemispheres. These areas do not correspond with classical vascular territories of the major cerebral arteries, because these show a large variation even in healthy subjects (Van der Zwan et al., 1992) – even more so in patients with MMV – and the PET technique did not allow identification of individual arterial territories. Per region, CVR scores were subjectively categorized as: −1: steal phenomenon visible (negative CVR in any part of that region), 0: no reactivity visible, 1: minimal reactivity, 2: normal reactivity (Fig. 2). The cerebellum (usually unaffected in MMV) was used as a reference for ‘normal reactivity’. In regions where large previous infarctions were detected on MRI, scoring of the CVR for the whole region was impossible so these regions were excluded from analysis. In case of the presence of smaller infarctions, only the CVR of the non-affected part of the region was scored. The same scoring was done for the follow-up scan, allowing the comparison of four CVR-score categories for each region over time. In addition, to appreciate qualitative changes in CVR subjectively, the blinded reviewers specifically looked at the changes in CVR between baseline and follow-up scans and rated those as either “improved”, “stable” or “deteriorated” for each region. For the comparison between operated patients and conservatively treated patients, the clinically most affected hemisphere of the conservatively patients was compared to the operated hemisphere of the surgical patients, while the least affected conservative hemisphere was compared to the contralateral hemisphere of the operative group.

### 2.4 Statistical analysis

For the PET study, the PET CVR scores per region were averaged per hemisphere for both conservative and operated patients. Any regions that could not be scored due to infarction were not included in the calculation of the average. The difference between before and after treatment was tested using a paired *t*-test (p < 0.05 was considered significant). For the TIA group, the difference in TIA frequency between pre and post-surgery were tested using the Wilcoxon signed-rank test. The figures and graphs were made with SPSS (IBM Corp, Version 26.0).

### 2.5 Contralateral TIA frequency

The frequency of TIAs that originated from the “contralateral” hemisphere – non-operated in the surgical group, or least affected in the conservative group - was noted by the treating neurologist or neurosurgeon, and were collected from the medical files. The last formal visit before operative treatment was used as the baseline time point. If patients received a contralateral operation within a year, the last out-patient clinic visit before second surgery was used as follow-up time point, otherwise the out-patient clinic visit around one year after the first operation was used as the time point of outcome measurement. In addition, the final available follow-up visit was compared to the baseline. TIA frequency was categorized as 0: no TIAs, 1: TIAs at least once per year, 2: TIAs at least once per month, 3: TIA’s at least once per week 4: TIAs at least once per day. TIAs could exist of (e.g.) transient weakness, numbness or loss of speech and were scored only when the treating physician had no doubt that the complaints were explained by ischemic events, and not caused by migraine or epilepsy. Furthermore, clinical and radiological infarction and hemorrhage was noted at baseline and follow-up.

## 3 Results

### 3.1 PET CVR results

Following the strict inclusion criteria for the assessment of changes in contralateral PET findings and TIA frequency, 12 bilateral MMV patients could be included for the PET study, of whom 7 were treated operatively. Five patients were first treated conservatively, of whom two were eventually operated (respectively 11 and 24 months after their first PET). These two were therefore included in both the conservative and operated group, leading to seven complete datasets – i.e. two sequential PET scans to compare – of both operated and conservatively treated patients. For patient and treatment characteristics see Table 1. All operated patients received a follow-up DSA after median 12 months (range 11–19). In all 6 patient with a direct procedure the bypass was open (100%). The indirect bypass of the only patient with an indirect procedure showed significant extra-cranial to intra-cranial contribution to the brain.

**Table 1:** Patient and treatment characteristics.

	PET study		TIA frequency study	
	Operative patients	Conservative patients	Operative patients	Conservative patients
Number of patients	7*	5	6	2
mean age at baseline	29 (SD 15, 10–53)	24 (SD 16, 7–41)	27 (SD 17, range 7–52)	34 (SD 13, range 24–43)
pediatric	5 (42%)	1 (14%)	2 (33%)	0 (0%)
Sex	5F (71%), 2 M (29%)	3F (60%), 2 M (40%)	5F (83%), 1 M (17%)	2F (100%)
type of bilateral MMV	3 MMD (50%), 3 MMS (50%)	3 MMD (60%), 2 MMS (40%)	6 MMD (100%)	2 MMD (100%)
presenting symptoms**:				
TIAs	6 (86%)	3 (60%)	6 (100%)	2 (100%)
infarction	2 (29%)	1 (20%)	5 (83%)	2 (100%)
hemorrhage	2 (29%)	2 (40%)	0 (0%)	0 (0%)
headache	3 (43%)	4 (80%)	1 (17%)	0 (0%)
cognitive decline	1 (14%)	1 (20%)	2 (33%)	0 (0%)
chorea	1 (14%)	0 (0%)	0 (0%)	0 (0%)
operation type:				
direct	1 (14%)	n/a	4 (67%)	n/a
indirect	1 (14%)	n/a	0	n/a
combined	5 (71%)	n/a	2 (33%)	n/a
most affected hemisphere	4 left, 3 right	2 left, 3 right	5 left, 1 right	2 right
second operation	0 (0%)	n/a	3 (50%)	n/a
median time from baseline to surgery (days)	11 (3–301)	n/a	11 (8–59)	n/a
median time from surgery to FU (days)	442 (361–483)	n/a	199 (143–366)	n/a
median time from baseline to FU for conservative treatment (days)	n/a	345 (274–469)	n/a	697 (329–1065)

Abbreviations: PET: Positron Emission Tomography, TIA: Transient Ischemic Attack, MMV: moyamoya vasculopathy, MMD: moyamoya disease, MMS: moyamoya syndrome. FU: follow-up. *)two of these patients were operated after initial conservative treatment and are included in both analyses. **)symptoms are not mutually exclusive.

**Fig. 3:**
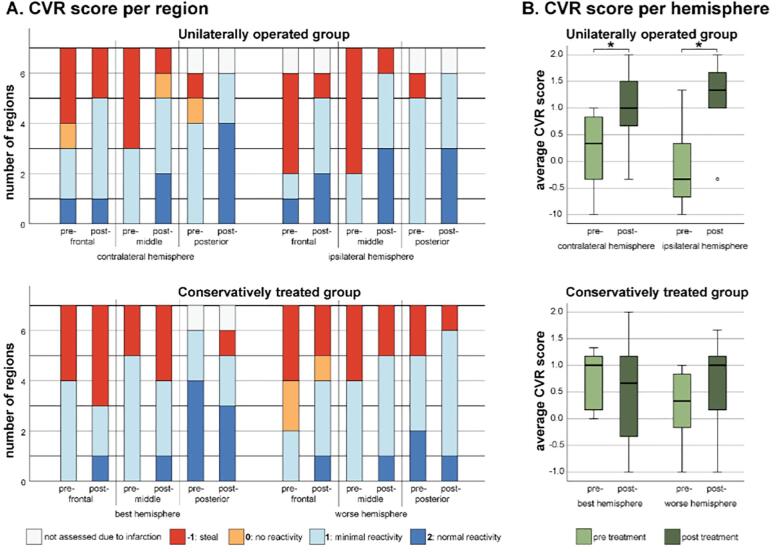
A. Counts of the pre-and post-operative PET CVR scores for each region for the operated patients, and counts of the pre- and post-follow-up PET CVR scores for the conservatively treated patients. B. Averaged PET CVR score per hemisphere pre- and post-treatment for both groups. *) significant difference (p < 0.05).

As shown in Fig. 3A, there was a postoperative reduction of the number of areas with unfavorable PET CVR scores (no reactivity or steal) and an increase of areas with favorable PET CVR scores (minimal or normal reactivity). This improvement was most prominent in the ipsilateral hemisphere, where the number of regions with steal decreased from 10 to 2 after surgery, and the number of regions with normal reactivity increased from 1 to 8 of the 19 scored regions in total (two regions could not be scored due to infarction). In the non-operated, contralateral hemisphere, an improvement was also seen, but to a lesser degree, with the number of steal regions decreasing from 8 to 3, and the number of regions with normal reactivity increasing from 1 to 7 (of a total of 20 scored regions). The average hemispherical CVR score increased for both hemispheres (Fig. 3B.). In the contralateral hemisphere the mean increased from 0.2 to 1.0 (p = 0.015) and in the ipsilateral hemisphere the mean increased from −0.1 to 1.2 (p = 0.012).

The conservatively treated group showed an opposite direction of changes. Over time, the clinically most affected hemisphere (comparable to the “ipsilateral” hemisphere of the operated patients) showed minor improvement in PET CVR scores, with a decrease in the number of regions with steal from 8 to 5, and an increase in regions with ‘normal reactivity’ from 2 to 3. The least affected hemisphere in conservative patients (comparable to the “contralateral” hemisphere of the operated patients) tended to deteriorate over time, with an increase in the number of steal regions from 5 to 8. The average hemispherical CVR score changed from a mean of 0.7 to 0.5 for the least affected hemisphere (p = 0.45, students T-test) and from 0.2 to 0.6 in the most affected hemisphere (p = 0.10).

**Fig. 4:**
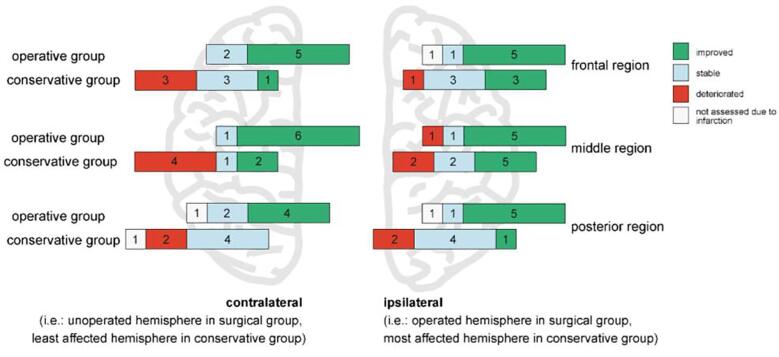
Subjective change in PET CVR scores, for the contralateral and ipsilateral hemisphere. Color bars representing the number of regions that showed either improved CVR (green), stable CVR (blue) or deteriorated CVR (red), as (blindly) assessed by visual comparison of first and second PET. (For interpretation of the references to color in this figure legend, the reader is referred to the web version of this article.)

Similarly, the subjectively assessed longitudinal change in PET CVR scores (Fig. 4) revealed that in the unilateral surgery group none of the 20 (0%) scored contralateral regions deteriorated and the majority of the regions (15 out of 20; 75%) improved. The other regions (5 out of 20, 25%) remained stable. In contrast, in the conservatively treated patients, PET CVR deteriorated in 9 of the 20 scored regions (45%) and improved in only 3 of the 20 scored regions (15%) in the clinically least affected (“contralateral”) hemisphere. The clinical follow-up is described in the supplementary materials.

### 3.2 TIA frequency results

**Fig. 5:**
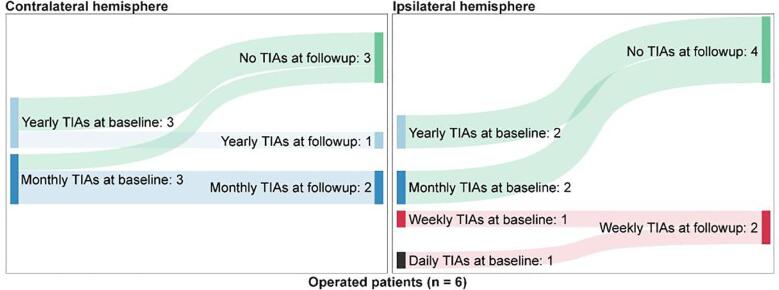
TIA frequencies before and after follow-up for the operated patients. The TIA frequencies are divided into different frequency categories (ranging from no TIAs to daily TIAs) and are compared pre- and postoperatively. The transition is graphically plotted using a Sankey-diagram. The numbers represent the number of patients.

Following the inclusion criteria for contralateral TIAs, eight patients were included after screening (see Fig. 1), of whom six underwent unilateral surgery and two were treated conservatively. A decrease in TIA frequency was seen in several patients after operation (Fig. 5) for both hemispheres: three patients did not experience TIAs originating from the contralateral hemisphere any more after operation, while three others remained stable in the TIA frequency from the contralateral hemisphere. For the ipsilateral hemisphere, five patients improved (of whom four did not have any TIAs after the first operation), while one patient remained stable. When statistically comparing the pre- and post-surgery scores TIAs originating from both hemispheres with the Wilcoxon Signed Ranks Test, the improvement of the TIAs originating from the ipsilateral hemisphere is significant (Z = -2.07, p = 0.038), while the improvement of the TIAs originating from the contralateral hemisphere does not reach significant levels (Z = -1.63, p = 0.102). Of the five patients who underwent an MRI in the first year after treatment, two patients showed new ischemic lesions in the ipsilateral hemisphere. No new infarctions were seen in the contralateral hemisphere. There were no patients with a new hemorrhage after follow-up. For the clinical follow-up of the operated and conservative treated patients please see the supplementary materials.

## 4 Discussion

We report the effect of unilateral revascularization on the contralateral hemisphere in bilateral MMV. Several patients showed improvement in the unoperated hemisphere after unilateral surgery, both radiologically (all 7 patients showed improvement in PET CVR score in one or more regions) and clinically (3 patients had improvement of TIA frequency, 3 remained stable, none deteriorated), though to a lesser extent than in the ipsilateral hemisphere. In conservatively treated patients, the least affected (“contralateral”) hemisphere did not show a similar pattern of improvement over time, suggesting that longitudinal changes in the operated group were explained by the surgical procedure, and not by the natural course of the disease. When assessing TIA frequencies at follow-up, we observed that the contralateral hemisphere of unilaterally operated patients improved in three out of six patients and remained stable in the remaining three. Considering the usually progressive nature of the disease, this is a remarkable effect. A possible explanation could be that by improving the hemodynamic status of the ipsilateral hemisphere the collateral demand from the contralateral hemisphere decreases, resulting in improvement of the hemodynamic status in regions of the contralateral hemisphere.

The ideal surgical strategy in patients with bilateral MMV has been a reason for debate for many years. Whenever possible, our approach is to perform a combined direct and indirect bypass. This is in contrast with other centers that advocate indirect procedures only (Blauwblomme et al., 2016). Similarly, some centers routinely treat both hemispheres in patients with bilateral MMV, either at one stage (Deng et al., 2017, Guzman et al., 2009, Kim et al., 2010), or in two separate procedures (Czabanka et al., 2016, Khan et al., 2003, Scott et al., 2004). Although the general consensus is that direct surgery leads to the fastest improvement of both CBF and CVR, definitive evidence favoring one approach over another is currently lacking. For pediatric patients, indirect surgery can be a technically easier alternative treatment, but may result in increasing the timeframe needed to form robust collateral circulation (Scott and Smith, 2009). A combined approach aims to provide fast revascularization by the direct bypass, while enlarging the area of revascularization on the longer term (Esposito et al., 2015).

In the light of this study, the effect of different surgical techniques on the contralateral hemisphere remains uncertain due to the small number of patients in each treatment group. To properly investigate this effect, a prospective study with CVR measurements before, and on multiple time points after the operation is required. However, such a study would be very difficult to execute considering the rarity and heterogeneity of the disorder.

Relatively few studies have addressed the effect of unilateral surgery on the contralateral hemisphere, and their results are not unequivocal. Some authors reported similar findings to ours. In a small study with 5 MMD patients, a significant postoperative increase in CBF (using Single Photon Emission Computed Tomography (SPECT) with an ACZ-challenge) in the contralateral frontal lobe was found after unilateral combined direct and indirect revascularization (Honda et al., 2006). It was hypothesized that the increase of contralateral CVR was associated with an unaffected anterior communicating artery. In another study, performed in 13 patients with MMD and 7 patients with other steno-occlusive diseases a significant increase in CVR in the vascular territories of the anterior and middle cerebral arteries and the cerebellum was found in both hemispheres after unilateral surgery (BOLD-CVR with CO_2_ inhalation) (Sam et al., 2015). In this study, possible anatomical variations of the circle of Willis did not seem to influence the improvement of the contralateral hemisphere. In another cohort, the MCA territory was investigated in 9 unilaterally operated MMD-patients using CT perfusion, before and after an ACZ challenge (Cheung et al., 2017). Both hemispheres appeared to benefit from the unilateral revascularization procedure although it is not clear how many of the unilateral operated patients were included in the CT-perfusion analysis.

Contrasting findings were reported by *Ma et al* (Ma et al., 2011), who examined 15 bilateral MMD patients with a unilateral clinical presentation. The patients received a Xenon-CT with ACZ challenge, before and 1, 3 and 6 months after unilateral surgery. A decrease of CVR in the contralateral hemisphere was reported 3 months after surgery, without mentioning CVR findings at 1 or 6 months postoperatively (Esposito et al., 2011). Another study of a heterogeneous group of 25 consecutive patients with intracranial steno-occlusive disease (including 12 MMV patients) looked at the effect of unilateral EC-IC bypass surgery on the BOLD-CVR (using a CO_2_-challenge) (Mandell et al., 2011). The authors reported a significant improvement in the ipsilateral, but not the contralateral hemisphere. Results of the subgroup of MMV patients were not reported separately, however. Furthermore, it is unclear whether the patients were all bilaterally or unilaterally affected. From the baseline CBF results, it seems the contralateral hemisphere in this group was less affected, making improvement after surgery more difficult to confirm.

To the best of our knowledge, no study has specifically investigated the change in clinical symptoms from the contralateral hemisphere after unilateral surgery in MMV. Apart from the hemodynamic studies described above, there is one report that described changes in the contralateral presence of the ‘ivy sign’ (leptomeningeal high signal intensity often seen on MRIs of MMV patients) (Ideguchi et al., 2011). Sixteen MMD patients, of whom 9 were operated unilaterally, were evaluated after STA-MCA bypass surgery, and of the 6 who had the ‘ivy sign’ before surgery in the contralateral hemisphere, this completely disappeared in 1 and decreased in 5 patients. Perfusion SPECT showed improvement in those areas. These findings support our results, however, the ‘ivy sign’ itself is poorly understood and its significance remains uncertain (Kronenburg et al., 2019).

The largest limitation of our study is its retrospective nature and thus a potential selection bias, resulting in a limited selection of patients suitable for investigating the research question (Fig. 1). Not all patients received a postoperative PET, and the selection of patients who did may have influenced the results. It can be argued that patients who recovered very well from unilateral surgery were less likely to receive a follow-up PET. On the other hand, severely affected patients are more likely to receive a contralateral operation, even without follow-up PET in between. The retrospective nature of this study also resulted in a lack of an optimal control group. We showed results of both the surgical and conservative group, but these treatments were not randomized but were specifically tailored to the patients. This treatment decision, per definition, hampers a proper comparison and drawing too strong conclusions. Another limitation was the small patient group. However, considering the rarity of the disease the present database is substantial, as in the Netherlands our hospital is the main reference center for MMV. Unfortunately, we could only identify two patients with TIAs originating from the least affected hemisphere to serve as a control group. This can be explained by the tendency – at least in our center – to consider most patients with bilateral TIAs candidates for revascularization.

Finally, scoring of the PET CVR score was done visually, by two reviewers who were blinded for the patients’ characteristics and outcomes. The [^15^O]H_2_O PET-CT with ACZ challenge is an excellent way of measuring CVR, which has proven to be a good indicator for both risks of infarction (Webster et al., 1995, Yonas et al., 1993) and success of treatment (Mandell et al., 2011), and is widely accepted for this purpose. However, while the ACZ challenge is widely used because of its ease of application, it does not always evoke the same response in patients due to (e.g.) variances in CVR for a given serum level (Fierstra et al., 2013). Furthermore, the technique used did not allow quantitative CBF evaluation. Scoring with a more automated, quantitative scoring protocol might have been more precise, but this was not possible with our available data. While the PET used in this study used ionizing radiation and was relatively expensive, newer less invasive CVR protocols using MRI (in combination with a hypercapnic (CO_2_) stimulus) have the potential to make routine careful monitoring easier, less invasive and possibly less burdensome for the patients in the future (Fan et al., 2019, Fierstra et al., 2018).

## 5 Conclusion

Contralateral CVR improvement and a decrease in the frequency of TIA’s originating from the contralateral hemisphere can occur after unilateral revascularization in bilateral MMV patients. In our cohort, all 7 patients showed improvement in PET CVR score in one or more contralateral regions, while 3 out of 6 patients improved in TIA frequency from the contralateral hemisphere and 3 remained stable. It is advisable to carefully monitor the CVR of bilateral MMV patients after a unilateral procedure, to determine the need for further, contralateral revascularization. In some patients, a second surgery over time, or a one-stage bilateral operation at onset, may prove unnecessary, thereby decreasing risks and treatment burden for patients.

## 6 Sources of Support

This work was supported by: Friends of WKZ/UMC Utrecht (1619144 / 1519131); Brain Technology Institute Foundation (1519131); the Dutch Brain Foundation ((2012(1)-179), the Christine Bader Fund Irene Children’s Hospital); the Tutein Nolthenius Oldenhof Fund and the Johanna Children Fund.

## CRediT authorship contribution statement

**Pieter T. Deckers:** Conceptualization, Methodology, Validation, Formal analysis, Investigation, Data curation, Writing - original draft, Visualization. **Wytse van Hoek:** Investigation, Visualization, Data curation, Writing - review & editing. **Annick Kronenburg:** Investigation, Writing - review & editing. **Maqsood Yaqub:** Resources, Writing - review & editing. **Jeroen C.W. Siero:** Writing - review & editing. **Alex A. Bhogal:** Conceptualization, Visualization, Supervision, Writing - review & editing. **Bart N.M. van Berckel:** Resources, Writing - review & editing. **Albert van der Zwan:** Resources, Funding acquisition, Supervision, Writing - review & editing. **Kees P.J. Braun:** Resources, Funding acquisition, Supervision, Writing - review & editing, Project administration, Methodology, Conceptualization.

## Declaration of Competing Interest

The authors declare that they have no known competing financial interests or personal relationships that could have appeared to influence the work reported in this paper.
